# A Genomewide Integrative Analysis of GWAS and eQTLs Data Identifies Multiple Genes and Gene Sets Associated with Obesity

**DOI:** 10.1155/2018/3848560

**Published:** 2018-05-08

**Authors:** Li Liu, Qianrui Fan, Feng Zhang, Xiong Guo, Xiao Liang, Yanan Du, Ping Li, Yan Wen, Jingcan Hao, Wenyu Wang, Awen He

**Affiliations:** ^1^School of Public Health, Health Science Center, Xi'an Jiaotong University, Xi'an, China; ^2^The First Affiliated Hospital, Xi'an Jiaotong University, Xi'an, China

## Abstract

To identify novel susceptibility genes and gene sets for obesity, we conducted a genomewide expression association analysis of obesity via integrating genomewide association study (GWAS) and expression quantitative trait loci (eQTLs) data. GWAS summary data of body mass index (BMI) and waist-to-hip ratio (WHR) was driven from a published study, totally involving 339,224 individuals. The eQTLs dataset (containing 927,753 eQTLs) was obtained from eQTLs meta-analysis of 5,311 subjects. Integrative analysis of GWAS and eQTLs data was conducted by SMR software. The SMR single gene analysis results were further subjected to gene set enrichment analysis (GSEA) for identifying obesity associated gene sets. A total of 13,311 annotated gene sets were analyzed in this study. SMR single gene analysis identified 20 BMI associated genes (*TUFM, SPI1, APOB48R*, etc.). Also 3 WHR associated genes were detected* (CPEB4*,* WARS2*, and* L3MBTL3)*. The significant association between Chr16p11 and BMI was observed by GSEA (FDR adjusted *p* value = 0.040). The TGCTGCT, MIR-15A, MIR-16, MIR-15B, MIR-195, MIR-424, and MIR-497 (FDR adjusted *p* value = 0.049) gene set appeared to be linked with WHR. Our results provide novel clues for the genetic mechanism studies of obesity. This study also illustrated the good performance of SMR for susceptibility gene mapping.

## 1. Introduction

Obesity is a complex health issue affecting millions of people around the world, causing serious health problems and a substantial economic burden in the developed country [1, 2]. It has been demonstrated that obesity was a major risk factor for multiple disorders, including cardiovascular diseases, diabetes mellitus type 2, and certain types of cancer [1, 3, 4]. Obesity is a multifactorial health problem in which genetic factors play an important role. A recent study showed SNPs contributing to adult BMI exert their effect at birth and in early childhood after investigating the contributions of 83 adult BMI associated SNPs on obesity-related traits in children from birth to 5 years [5]. Extensive genetic association studies have been conducted and identified multiple BMI, WHR, or other obesity traits associated genes [6, 7]. For instance, Loos found that a variant near* CREBRF* was associated with increased risk of obesity while being associated with decreased risk of type 2 diabetes in 3000 Samoans [8]. Associations between body mass index and ~2.8 million SNPs in up to 123,865 individuals were analyzed. Then 42 SNPs were followed up in 125,931 additional individuals. 18 new loci associated with BMI were identified, including GPRC5B [9]. Another study indicated the genetic variants in the FTO gene were associated with BMI, hip circumference, and body weight via a whole genome association scan for these three obesity-related quantitative traits [10]. However, despite the considerable efforts, the genetic mechanism of obesity remains elusive now. The heritability explained by the identified loci was limited, suggesting the existence of undiscovered susceptibility loci for obesity.

Genomewide association study (GWAS) contributes greatly to the susceptibility gene mapping of human complex diseases. Despite the great power of GWAS, it has several limitations. For instance, due to strict genomewide significance thresholds, GWAS usually focuses on the most significant loci and ignores the rest with moderate and modest phenotypic effects [11, 12]. Without considering biological interactions among genes, the susceptibility genes identified by GWAS are usually functionally independent, providing limited information for clarifying the genetic mechanism of target diseases [12]. Inspired by the gene set analysis of microarray data, GWAS-based gene set association analysis was proposed [13]. Gene set association analysis can gain additional insight into the genetic mechanism of complex diseases, through integrating GWAS results and prior functional information of biological gene sets [14].

Expression quantitative trait loci (eQTLs) are generally noncoding loci that influence the levels of gene transcripts [15]. With the increased genomewide eQTLs resources [16–18], more and more attention is paid to reveal the biological function of eQTLs. Recent studies have confirmed the important roles of eQTLs in the development of human complex diseases [19, 20], including obesity [21]. For example, Nicolae et al. found that disease-associated loci are significantly enriched in eQTLs [22].

Recently, summary data-based Mendelian randomization (SMR) was proposed [23]. SMR resembles a Mendelian randomization that regards genetic variant as an instrumental variable to evaluate the association between gene expression and target traits. SMR is capable of integrating GWAS summary data and eQTLs annotation information to identify novel causal genes, the expression levels of which are associated with complex diseases [23]. SMR shows a high power for identifying novel susceptibility genes of complex diseases [23].

In this study, utilizing a recently published eQTLs annotation dataset, SMR was first applied to large scale GWAS summary data for screening novel genes, the expression levels of which were associated with obesity. To reveal the biological significance of identified genes, we also extended the SMR approach and conducted a genomewide gene set expression association analysis of obesity. The SMR single gene summary data was subjected to gene set enrichment analysis (GSEA) to identify obesity associated biological gene sets. The main objective of our study is to reveal obesity associated novel genes and gene sets on expression levels.

## 2. Methods

### 2.1. GWAS Summary Datasets

Large scale GWAS meta-analysis summary data of body mass index (BMI) and waist-to-hip ratio (WHR) was used here [24]. Briefly, this GWAS dataset totally contained 339,224 individuals (322,154 European descents and 17,072 non-European descents) from 125 studies, including 82 with GWAS results (*n* = 236,231) and 43 with Metabochip results (*n* = 103,047). European-descent genotype imputation was conducted using the HapMap CEU reference panel, while the African American and Hispanic genotype imputation was conducted using HapMap CEU + YRI + CHB + JPT reference panel [25]. The inverse variance-weighted method implemented in METAL was used to perform fixed effects meta-analyses [26]. Detailed information of study subjects, experimental designs, and statistical analysis is available in the published study [24].

### 2.2. SMR Single Gene Analysis

The GWAS meta-analysis summary datasets of BMI and WHR were subjected to SMR analysis [23]. The eQTLs annotation dataset established by Westra et al. was applied here [27]. This eQTLs dataset was identified in blood samples from 5,311 individuals and replicated in another sample of 2,755 individuals. Illumina gene expression arrays were used for gene expression analysis. SNP genotype data was imputed against HapMap 2 reference panels. 923,021 cis-eQTLs for 14,329 gene expression probes and 4,732 trans-eQTLs for 2,612 gene expression probes were identified at false discovery rate (FDR) < 0.05. Significant genes were identified at SMR *p* values < 9.28 × 10^−6^ (0.05/5390) after Bonferroni correction.

### 2.3. Gene Set Enrichment Analysis

The SMR gene-level summary data of BMI and WHR were analyzed by GSEA for gene set association analysis [13]. The latest gene set-gene annotation database (msigdb.v5.1) was downloaded from the GSEA Molecular Signatures Database (http://software.broadinstitute.org/gsea/msigdb/index.jsp). It contained a total of 13,311 annotated gene sets [28]. 5,000 permutations were conducted to calculate the empirical *p* value and FDR adjusted *p* value of each gene set [13]. Significant gene sets were identified at FDR adjusted *p* values < 0.05.

## 3. Results

### 3.1. Single Gene Analysis

A total of 5,390 genes with both GWAS summary and eQTLs data were analyzed by SMR in this study. After strict Bonferroni correction, SMR identified 20 BMI associated genes (Table 1), such as TUFM (FDR adjusted *p* value = 3.16 × 10^−22^), SPI1 (FDR adjusted *p* value = 2.83 × 10^−12^), and APOB48R (FDR adjusted *p* value = 4.13 × 10^−12^). For WHR, SMR detected 3 significant genes, including CPEB4 (FDR adjusted *p* value = 5.48 × 10^−12^), WARS2 (FDR adjusted *p* value = 2.21 × 10^−8^), and L3MBTL3 (FDR adjusted *p* value = 9.06 × 10^−6^).

### 3.2. Gene Set Enrichment Analysis

We found that the Chr16p11 (*p* value = 1 × 10^−3^) gene set and NITROGEN_COMPOUND_METABOLIC_PROCESS (*p* value = 1 × 10^−3^) were significantly associated with BMI. For WHR, the TGCTGCT, MIR-15A, MIR-16, MIR-15B, MIR-195, MIR-424, MIR-497 (FDR adjusted *p* value = 0.049) gene set showed significant association signal.

## 4. Discussion

GWAS plays a critical role in the susceptibility gene mapping of complex diseases. In spite of its great power, a large part of GWAS identified loci located in noncoding chromosomal regions, making it challenging to elucidate the roles of these identified loci in the development of target diseases. Recent studies demonstrated the significant impact of eQTLs on the disease development through regulating the expression of disease-associated genes. To better illuminate the genetic basis of obesity, utilizing the latest SMR approach and published GWAS summary data, we conducted an eQTLs-based single gene expression association analysis and gene set enrichment analysis for obesity. A group of genes and gene sets were identified to be significantly associated with obesity.

SMR single gene analysis found that TUFM was the most significant gene associated with BMI. TUFM encodes the Tu translation elongation factor, Mitochondrial, which participates in mitochondrial protein translation. TUFM mutation can result in dysfunction of oxidative phosphorylation and lead to lactic acidosis and fatal encephalopathy [29]. Gutierrez-Aguilar et al. observed that TUFM was significantly upregulated in adipose tissue and liver in high-fat diet rat [30]. Another notable gene was APOBR, encoding apolipoprotein B48 receptor. A previous study found that high-fat meal increased the transcriptional activity of APOBR in circulating monocytes [31]. The MYBPC3 gene encodes the cardiac myosin binding protein C (MyBP-C), which is found in heart (cardiac) muscle cells. Previous studies showed that mutations in MYBPC3 genes are one of the most common genetic causes of hypertrophic cardiomyopathy (HCM) [32, 33]. More studies are warranted to clarify the potential mechanism underlying the associations between MYBPC3 and obesity. Our study also found another relatively significant variant SPI1, a protein coding gene. Lane et al. revealed that the level of SPI1 gene affected adipose generation [34].

*CPEB4* encodes cytoplasmic polyadenylation element binding protein 4. The SMR analysis result suggests that the expression level of CPEB4 was significantly associated with WHR. Our result is consistent with that of a previous genetic meta-analysis, which observed significant associations between CPEB4 and WHR [35]. Additionally, SMR analysis detected significant association signals for WARS2 and L3MBTL3. WARS2 encodes tryptophanyl TRNA synthetase 2, Mitochondrial, while L3MBTL3 encodes L (3) Mbt-Like 3 (*Drosophila*). The biological functional studies of WARS2 and L3MBTL3 are limited. To the best of our knowledge, little effort has been paid to investigate the possible impact of WARS2 and L3MBTL3 on the development of obesity. Further studies are warranted to confirm our findings and to reveal the potential mechanism of identified genes involved in the development of obesity.

Several significant gene sets were identified via gene set enrichment analysis, including chr16p11 and NITROGEN_COMPOUND_METABOLIC_PROCESS for BMI and TGCTGCT, MIR-15A, MIR-16, MIR-15B, MIR-195, MIR-424, and MIR-497 for WHR. The Chr16p11 gene set consists of 212 genes, some of which are associated with obesity, including SH2B1, APOBR, SULT1A1, SULT1A2, and TUFM. Volckmar et al. identified variants in APOBR and SH2B1 which are associated with extreme obesity in the chromosomal region chr16p11.2 [36]. It was reported that the deletions of 16p11.2 SH2B1-containing region were pathogenic and associated with developmental delay as well as obesity via an array comparative hybridization on 23,084 patients in a clinical setting [37]. SH2B1 was one of the genes in this gene set, which was demonstrated to be associated with obesity. Multiple isoforms of SH2B1 (alpha, beta, gamma, and/or delta) were expressed in numerous tissues, including the brain, liver, muscle, and adipose. Previous study suggested that SH2B1 was essential for regulating energy balance, body weight, peripheral insulin sensitivity, and glucose homeostasis [38, 39]. Another significant gene set identified for BMI was NITROGEN_COMPOUND_METABOLIC_PROCESS. Several genes among this gene set were associated with obesity, including GNPDA2, MTHFR, and ATF4 [40, 41].

The TGCTGCT, MIR-15A, MIR-16, MIR-15B, MIR-195, MIR-424, and MIR-497 gene set mainly comprises miR-15 microRNA precursor family, including MIR-15A, MIR-15B, MIR-16, MIR-195, and MIR-497. Dong et al. found that MIR-15A and MIR-15B could promote adipogenesis in porcine preadipocyte [42]. Perri et al. observed that MIR-15A was significantly upregulated in obesity patients [43]. Our results support the important role of miR-15 microRNA precursor family in the pathogenesis of obesity.

The significant genes identified by SMR were also significant in the original GWAS, which illustrated the good performance of SMR for susceptibility gene mapping. SMR is capable of integrating GWAS summaries and eQTLs annotation information to detect the genes, whose expression levels are associated with complex diseases. The SMR results were consistent with that of previous functional studies of obesity and highlighted several genes for obesity. Therefore, our study results suggested that the genes identified by SMR contributed to the development of obesity through abnormal expression of the genes. Furthermore, we extended SMR to gene set enrichment analysis for obesity, and finally several significant gene sets associated with BMI and WHR were identified, providing novel clues on the genetic mechanism of obesity. That is exactly the extension and innovation of our study to SMR analysis.

In summary, this study explored the genetic basis of obesity via integrating analysis of GWAS and eQTLs data utilizing SMR approach. We identified multiple novel genes and gene sets, which were significantly associated with BMI or WHR. Our results provide new clues for clarifying the genetic mechanism of obesity. This study also illustrated the good performance of SMR and extended it to gene set association analysis for complex diseases.

## Figures and Tables

**Table 1:** List of significant genes identified by SMR.

	Gene	Top SNP	MAF	GWAS		SMR	
				*β*	*p* value	*β*	*p* value
BMI	TUFM	rs2008514	0.386	0.031	1.46 × 10^−23^	0.034	3.16 × 10^−22^
	SPI1	rs11039212	0.670	0.023	8.75 × 10^−13^	−0.028	2.83 × 10^−12^
	APOB48R	rs1968752	0.359	0.027	4.69 × 10^−17^	0.106	4.13 × 10^−12^
	MYBPC3	rs11039212	0.670	0.023	8.75 × 10^−13^	−0.036	4.60 × 10^−12^
	SPNS1	rs8045689	0.295	0.021	1.17 × 10^−10^	0.024	1.52 × 10^−10^
	MAP2K5	rs4331293	0.258	−0.025	2.24 × 10^−13^	0.091	1.92 × 10^−10^
	FNBP4	rs10838774	0.458	−0.021	6.73 × 10^−13^	0.126	2.51 × 10^−8^
	NT5C2	rs11191580	0.092	0.031	2.59 × 10^−9^	0.058	2.62 × 10^−8^
	HS.371060	rs4704221	0.391	−0.018	1.56 × 10^−8^	0.044	3.08 × 10^−8^
	GTF3A	rs7988412	0.187	0.026	1.23 × 10^−8^	−0.054	4.02 × 10^−8^
	SBK1	rs4788076	0.354	0.025	2.16 × 10^−17^	−0.192	1.89 × 10^−7^
	C18ORF8	rs1788821	0.338	0.021	1.89 × 10^−8^	−0.080	2.48 × 10^−7^
	C5ORF37	rs17649266	0.210	−0.020	2.70 × 10^−8^	−0.064	4.28 × 10^−7^
	NPC1	rs1624695	0.329	0.021	2.21 × 10^−8^	0.111	1.66 × 10^−6^
	ADCY3	rs6737082	0.462	0.030	2.10 × 10^−16^	−0.267	1.80 × 10^−6^
	ARL3	rs12415043	0.095	0.026	4.07 × 10^−7^	0.060	1.97 × 10^−6^
	L3MBTL3	rs6569648	0.228	0.017	1.84 × 10^−6^	0.029	2.30 × 10^−6^
	SAE1	rs8101149	0.280	−0.025	7.75 × 10^−10^	−0.157	2.46 × 10^−6^
	CUGBP1	rs12419692	0.363	0.024	1.56 × 10^−14^	0.202	4.05 × 10^−6^
	ZNF668	rs11150604	0.377	−0.017	4.34 × 10^−6^	−0.043	7.29 × 10^−6^

WHR	CPEB4	rs966544	0.314	0.025	2.3 × 10^−12^	0.026	5.48 × 10^−12^
	WARS2	rs10923698	0.142	−0.036	4.0 × 10^−9^	−0.076	2.21 × 10^−8^
	L3MBTL3	rs6569648	0.228	−0.018	3.6 × 10^−6^	−0.031	9.06 × 10^−6^

## Abbreviations

GWAS: Genomewide association study
eQTLs: Expression quantitative trait loci
BMI: Body mass index
WHR: Waist-to-hip ratio
GSEA: Gene set enrichment analysis
SMR: Summary data-based Mendelian randomization
FDR: False discovery rate.
